# Metabolomic Profiling of Respiratory Muscles and Lung in Response to Long-Term Controlled Mechanical Ventilation

**DOI:** 10.3389/fcell.2022.849973

**Published:** 2022-03-22

**Authors:** Ya Wen, Xiang Zhang, Lars Larsson

**Affiliations:** ^1^ Department of Physiology and Pharmacology, Karolinska Institutet, Bioclinicum, Stockholm, Sweden; ^2^ Department of Molecular Medicine and Surgery, Karolinska Institutet, Bioclinicum, Stockholm, Sweden; ^3^ Department of Clinical Neuroscience, Karolinska Institutet, Bioclinicum, Stockholm, Sweden

**Keywords:** controlled mechanical ventilation, critical illness myopathy, ventilator-induced diaphragm dysfunction, respiratory muscles, lung, metabolomics

## Abstract

Critical illness myopathy (CIM) and ventilator-induced diaphragm dysfunction (VIDD) are characterized by severe muscle wasting, muscle paresis, and extubation failure with subsequent increased medical costs and mortality/morbidity rates in intensive care unit (ICU) patients. These negative effects in response to modern critical care have received increasing attention, especially during the current COVID-19 pandemic. Based on experimental and clinical studies from our group, it has been hypothesized that the ventilator-induced lung injury (VILI) and the release of factors systemically play a significant role in the pathogenesis of CIM and VIDD. Our previous experimental/clinical studies have focused on gene/protein expression and the effects on muscle structure and regulation of muscle contraction at the cell and motor protein levels. In the present study, we have extended our interest to alterations at the metabolomic level. An untargeted metabolomics approach was undertaken to study two respiratory muscles (diaphragm and intercostal muscle) and lung tissue in rats exposed to five days controlled mechanical ventilation (CMV). Metabolomic profiles in diaphragm, intercostal muscles and lung tissue were dramatically altered in response to CMV, most metabolites of which belongs to lipids and amino acids. Some metabolites may possess important biofunctions and play essential roles in the metabolic alterations, such as pyruvate, citrate, S-adenosylhomocysteine, alpha-ketoglutarate, glycerol, and cysteine. Metabolic pathway enrichment analysis identified pathway signatures of each tissue, such as decreased metabolites of dipeptides in diaphragm, increased metabolites of branch-chain amino acid metabolism and purine metabolism in intercostals, and increased metabolites of fatty acid metabolism in lung tissue. These metabolite alterations may be associated with an accelerated myofibrillar protein degradation in the two respiratory muscles, an active inflammatory response in all tissues, an attenuated energy production in two respiratory muscles, and enhanced energy production in lung. These results will lay the basis for future clinical studies in ICU patients and hopefully the discovery of biomarkers in early diagnosis and monitoring, as well as the identification of future therapeutic targets.

## Introduction

Intensive care and intensive care units (ICUs) have undergone significant development in the past 65 years along with the improvements of medical technology, therapeutics, and basic understanding of pathophysiology, resulting in improved survival. However, lifesaving ICU interventions are associated with complications having negative consequences for morbidity, mortality, and health care costs (Friedrich et al., 2015). Critical illness myopathy (CIM) is observed in ∼30% of the general ICU population exposed to long-term mechanical ventilation and almost 100% in some sub-populations of ICU patients (e.g., neuro-ICU patients exposed to long-term controlled mechanical ventilation) (Llano-Diez et al., 2012; Cacciani et al., 2022) and is characterized by severe muscle wasting, paralysis of limb/trunk muscles, and a preferential myosin loss while craniofacial muscles are spared or less affected. CIM is associated with increased mortality/morbidity and 3-fold higher ICU costs (not including post-ICU costs) (Larsson, 2008; Friedrich et al., 2015). Mechanical ventilation is a lifesaving treatment and ∼40% of ICU patients are mechanically ventilated for a median duration of 5–7 days, but weaning from mechanical ventilation is a time-consuming process, comprising ∼40% of the time spent on the ventilator (Larsson and Friedrich, 2016). Additional problems in weaning are observed in 20–30% of these patients due to ventilator induced diaphragm muscle dysfunction (VIDD), resulting in prolonged intensive care and increased risk of secondary pulmonary complications and mortality. Signs of VIDD have been reported early within the first day of mechanical ventilation in both clinical and experimental studies (Jaber et al., 2009; Powers et al., 2013; Corpeno et al., 2014; Tang et al., 2017). In contrast to limb muscles and intercostals, there is no preferential myosin loss in the diaphragm but myosin post-translational modifications have a significant negative effect on diaphragm force-generating capacity (Corpeno et al., 2014; Salah et al., 2016). In limb muscles, myosin function is also negatively affected by post-translational modifications at an early stage of mechanical ventilation, preceding the myosin loss which is the major factor underlying the dramatically declined limb muscle force-generation capacity (Cacciani et al., 2020). The mechanisms causing VIDD are considered intrinsic to diaphragm muscle fibers, rather than related to alterations in the lungs, thorax/abdominal compliance, or neural input (Jaber et al., 2011; Powers et al., 2013). These patients account for ∼30% of overall ICU costs or a staggering $64 billion in the US annually (Zilberberg and Shorr, 2008) prior to the COVID-19 pandemic. Approximately two thirds of critically ill COVID-19 receive mechanical ventilation within 24 h of admission with many being mechanically ventilated for 12 days or longer (Grasselli et al., 2021). The ventilator induced lung injury (VILI) is characterized by structural changes in lung tissue, reduced gas exchange capacity and ventilatory capability (Griffiths and Finney, 2011; Marini and Gattinoni, 2020). In the general ICU population, up to 50% of mechanically ventilated patients develop an acute lung injury (Griffiths and Finney, 2011). In the experimental ICU model used in the current study, histopathological signs of VILI may be observed already within 12 h of controlled mechanical ventilation (CMV).

The mechanisms underlying the differences in myosin loss between diaphragm and limb/intercostal muscles are not fully understood but has been suggested to be due to intrinsic differences related to embryological origins, innervation, and activation patterns (Hirasawa and Kuratani, 2013; Corpeno et al., 2014). In addition, the complete mechanical silencing of limb muscles may contribute to the muscle specific differences in myosin loss since mild passive mechanical loading reduced the myosin loss in distal hind limb muscles (Renaud et al., 2013). The more than 11-fold larger number of passive mechanical loadings of the diaphragm by the ventilator may accordingly contribute to the muscle specific differences in myosin expression, especially since the mechanical strain on the intercostals is negligible compared to the diaphragm during CMV in the experimental model used in this study.

Neuromuscular blockers, corticosteroids and sepsis have been proposed as major factors triggering CIM, but CIM is observed in the absence of any of these factors. The only two factors ICU patients with CIM have in common are long-term mechanical ventilation and immobilization, i.e., absence of weight bearing and the internal strain caused by activation of contractile proteins (Ochala et al., 2011). In the general ICU population, assisted mechanical ventilation is the preferred mode of ventilation since it induces less ventilator induced lung injury (VILI) than controlled mechanical ventilation, but CMV is used in patients pharmacologically paralyzed or when there is an insufficient central ventilatory drive such as in many neuro-ICU patients, or in COVID-19 patients ventilated in the prone position. In our previous studies on neuro-ICU patients exposed to long-term CMV, all patients developed the CIM pheno-/geno-type (Llano-Diez et al., 2012). This is corroborated by our experimental studies in which CMV for a period of 5 days and longer consistently led to CIM with a preferential myosin loss, transcriptional downregulation of myosin synthesis, cytokine activation, and systemic inflammation (Norman et al., 2006; Llano-Diez et al., 2011; Ochala et al., 2011; Renaud et al., 2013; Corpeno Kalamgi et al., 2016; Kalamgi and Larsson, 2016; Akkad et al., 2019). In the diaphragm, long-term CMV also induced the activation of protein degradation pathways, post-translational protein modifications, impaired mitochondria structure/function, dramatic impairments in contractile properties at the cell and motor protein levels, as well as downregulated energy production, but no transcriptional downregulation of myosin synthesis and no preferential myosin loss (Dworkin and Dworkin, 2004; Corpeno et al., 2014; Salah et al., 2016).

In addition to generating force and movement, skeletal muscle also has multiple other functions such as being a hormone producer, passive protector of bone and inner organs, as well as a metabolic organ and amino acid reservoir (Argilés et al., 2016). Skeletal muscle metabolomic profile is accordingly of significant interest in both health and disease with metabolites representing the end products of alterations at the gene and protein levels in response to external and internal stimuli (Jacob et al., 2019). We have therefore conducted an untargeted metabolomics approach using a unique experimental ICU model where rats have been exposed to 5 days CMV to obtain a global view on the metabolite alterations in response to long-term CMV to improve our understanding of VILI, VIDD and CIM pathogenesis, and the identification of biomarkers and therapy targets. The two respiratory muscles, diaphragm and intercostals, have been analyzed to represent muscles strongly associated with VIDD and CIM, respectively.

## Materials and Methods

### Animals

Female 280–350 g Sprague Dawley rats were exposed to deep sedation with isoflurane, controlled mechanical ventilation and post-synaptic neuromuscular blockade with α-cobrotoxin for 5 days (*n* = 5) and compared with sham operated control rats (*n* = 5). All aspects of this study were approved by the ethical committees at Karolinska Institute and Uppsala University.

### Experimental ICU Model

All experimental animals were maintained in fluid and nutritional balance throughout the duration of the experimental procedures by introducing: 1) intra-arterial solution (0.6 ml/h) containing 21 ml H_2_O, 24 ml 0.5 N lactated Ringer, 0.84 g oxacillin Na, 0.65 mg α-cobrotoxin, 0.3 mg vitamin K (Synkavite), 20 meq K^+^ (as KCl); 2) an intra-venous solution (0.6 ml/h) containing 26 ml H_2_O, 16 ml 0.5 N lactated Ringer, 20% glucose (Baxter, Deerfield, IL, United States), 0.32 g oxacillin Na for the initial 24, then 8.5% Travasol amino acids (Baxter) and 20% Intralipid (Kabi, Uppsala, Sweden) were added subsequently to provide adequate nutrients (Dworkin and Dworkin, 2004). Body temperature, peripheral perfusion, and oxygen saturation [measured continuously with an infrared probe in a hind limb paw, MouseSTAT (Kent Scientific corp., Torrington, CT, United States)] were monitored and maintained in the physiological range. The sham-operated controls were anesthetized with isoflurane, maintained in spontaneous breathing, received intravenous and intra-arterial solutions, and sacrificed within 2 h of the initial isoflurane anaesthesia and surgery.

During surgery or any possible irritating manipulation, the anaesthetic isoflurane level, i.e., the Minimum Alveolar Concentration (MAC) was >1.5%, which maintains the following states: 1) the electroencephalogram (EEG) was synchronized and dominated by high-voltage slow-wave activity; 2) mean arterial pressure, 90–100 mmHg, heart rate maintained below 420 beats/min; and 3) no evident EEG, blood pressure or heart rate responses to surgical manipulation. Isoflurane was delivered into the inspiratory gas stream by a precision mass-flow controller. After the initial surgery, isoflurane was gradually lowered (over 1–2 days) and maintained at MAC <0.5% during the remaining experimental period. Rats were ventilated through a coaxial tracheal cannula at 72 breaths/min with an inspiratory and expiratory ratio of 1:2 and a minute volume of 180–200 ml and gas concentrations of 40% O_2_, 56.5% N_2_, and 3% CO_2_, delivered by a precision (volume drift <1%/wk) volumetric respirator. Airways pressure was monitored continuously as well as end-tidal CO_2_ (EtCO_2_) and normocapnic condition maintained (EtCO_2_ = 37–45 mmHg) as well as normoxia (SpO_2_ > 90%). Intermittent hyperinflations (6 per hour at 19–20 cm H_2_O) over a constant positive end-expiratory pressure (PEEP = 1.5 cm H_2_O) were set to prevent atelectases. Post-synaptic neuromuscular blockade was induced on the first day (150 µg α-cobrotoxin) and maintained by continuous infusion (187 µg/day). Mechanical ventilation started after the neuromuscular blockade induction avoiding hypercapnia and hypoxemia. Experiments were terminated after 5 days. Female rats were preferred because easier urine bladder catheterization for diuresis monitoring. The diuresis was maintained above 1 ml/h. In no case did animals show any signs of infections or septicaemia. Animals were euthanized under deep isoflurane anaesthesia by removal of the heart.

Immediately after rats had been euthanized, lungs, diaphragm, and intercostal muscles were gently dissected and snap frozen in liquid propane chilled by liquid nitrogen and stored at −140°C until metabolomic analyses. The midcostal part of the diaphragm and all three layers of intercostal muscles between the 6th and 8th intercostal spaces from sternum to the mid-clavicular region were chosen for analyses. The right lung was chosen for analyses of pulmonary tissue.

### Metabolomic Profiling

Dissected diaphragm, intercostal muscles and lung tissue of both experimental and control groups were shipped on dry ice to Metabolon (Durham, North Carolina). When arriving, all samples were immediately logged into Metabolon LIMS system and stored at −80°C until processed. The metabolite extractions were prepared using the automated MicroLab STAR® system from Hamilton Company. Several recovery standards were added prior to the first step in the extraction process for quality control purpose. After removing proteins and organic solvent, the resulting extract was stored overnight in liquid nitrogen before subject to untargeted metabolomics profiling using ultra-high performance liquid chromatography-tandem mass spectroscopy (UPLC-MS/MS).

### Identification of Differential Metabolites

Raw peak area data, generated by the metabolomics platform (Metabolon, Durham, North Carolina, United States), of all samples were subject to quality control. Firstly, raw peak area data was subject to batch-normalization to remove batch effect. For each metabolite, the raw values in the experimental samples were divided by the median of those samples in each instrument batch, giving each batch and thus the metabolite a median of one. For each metabolite, the missing values were imputed as the minimum value across all batches in the median scaled data. Subsequently, the batch-normalized-imputed data were log-transformed, which typically displayed a normal distribution and was used in subsequent statistical analyses. Each metabolite was annotated by Metabolon, including classification and pathways. One lung sample obtained after 5-day CMV was abnormal according to analysis from Metabolon technician and was removed from subsequent analyses. After quality control, principal component analysis was performed using PCAtools to obtain an overview on the effect of 5-day CMV on the metabolite profiles in each tissue, in the meanwhile, differential analysis was performed by general linear model using lmerTest R package (Kuznetsova et al., 2017). Metabolites with *p*-value < 0.05 and absolute fold change ≥1.5 were assigned as differential metabolites (DMs). All differential metabolites in each tissue were subject to hierarchical clustering analysis by pheatmap R package.

### Metabolite-Metabolite Interaction (MMI) Network Analysis

Identified DMs in each tissue were subject to MMI network analysis by MetaboAnalyst 5.0 (Pang et al., 2021), respectively. The interactions among metabolites were indicated by chemical-chemical association which is based on and extracted from STITCH database (Kuhn et al., 2008), but only highly confident interactions were used in the network. Metabolites with the highest betweenness/association with other metabolites were considered as metabolite hubs and highlighted in the network.

### Metabolic Pathway Enrichment Analysis

Identified DMs in each tissue were subject to metabolic pathway enrichment analysis. Enriched pathway with DMs number ≥5 was evaluated by several methods including zscore (Lyu et al., 2021), enrichment value, and metabolite set enrichment analysis (MSEA). Zscore was used to indicate the down-/up-regulated status of the enriched pathway.

$$Zscore=\left(up-down\right)/\sqrt{count}$$

*count* is the number of DMs assigned to an enriched term, whereas *up* and *down* are the number of assigned DMs increased or decreased, respectively. Enrichment value (provided by Metabolon) was also employed in the present study to indicate the reliability of the enriched pathway. It was calculated as the following,

$$Enrichment\;Value=\left(k/m\right)/\left(\frac{n-k}{N-m}\right)$$

*k* represents the number of DMs in the pathway, *m* represents the number of detected named metabolites in the pathway, *n* represents the number of DMs in all pathways, and *N* represents the number of detected named metabolites in all pathways. The enriched pathways are considered as the metabolic pathway signatures of each tissue when meeting the cutoff absolute (abs) zscore ≥2.5 and enrichment value ≥1.5.

On the other hand, identified DMs in each tissue were subject to MSEA, respectively, using the annotation list of all detected metabolites in each tissue as the background. MSEA is a method supposed to facilitate the identification and interpretation of changes in metabolite concentration patterns in a biologically meaningful way (Pang et al., 2021).

Pathways were considered as significantly enriched when *p* < 0.05. The enrichment results were compared among tissues to reveal similarities and differences.

### Statistics

All statistical analyses were performed in R statistical computing software version 4.1. Specifically, PCAtools was for principal component analysis, lmerTest was for differential analysis, pheatmap was for hierarchical clustering analysis, and MetaboAnalyst 5.0 was for MMI network analysis and MSEA. *p*-value < 0.05 represents a statistically significant difference.

## Results

### Alterations in Metabolomic Profiles in Response to 5-day CMV

**FIGURE 1:**
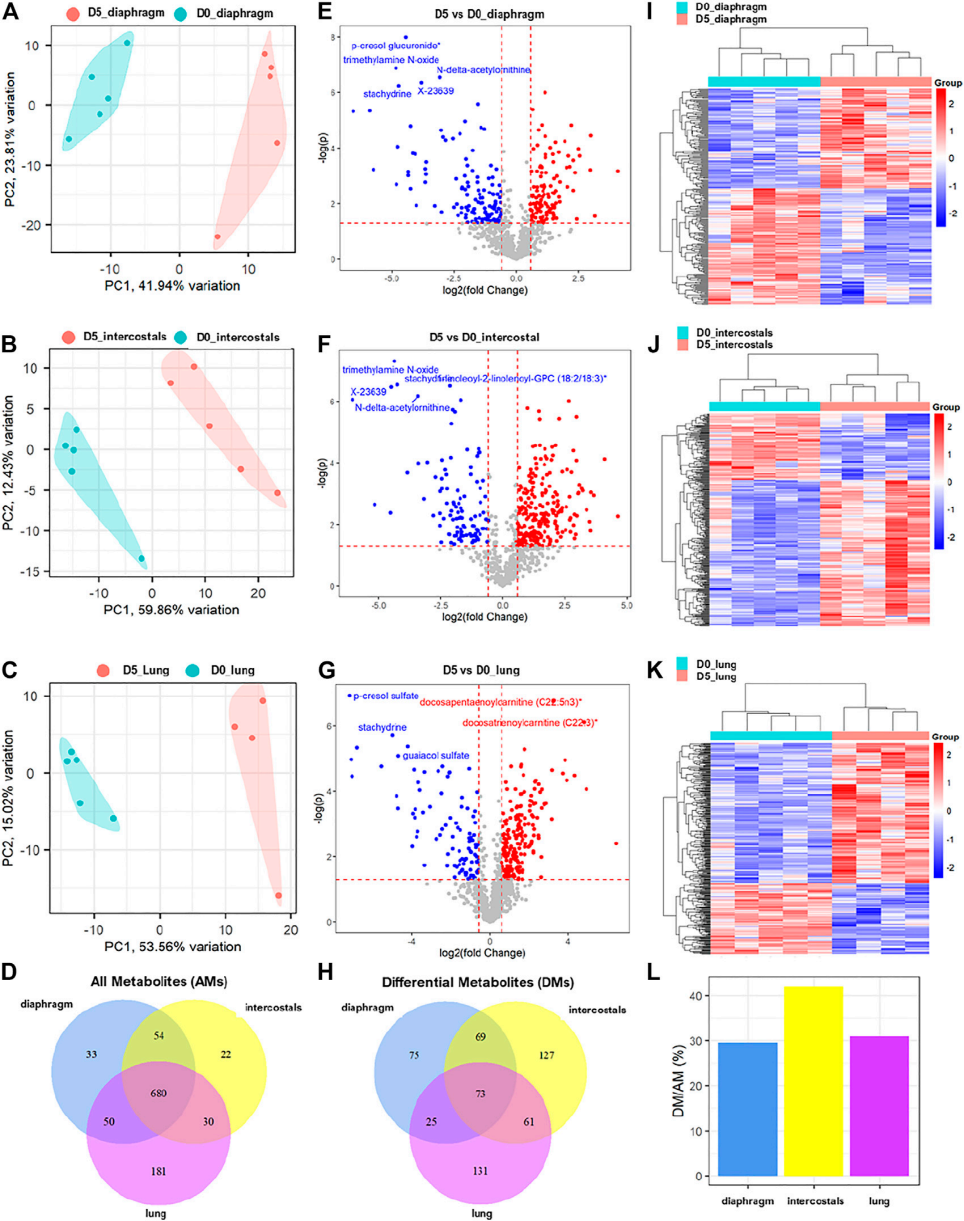
The alterations of metabolomic profiles in diaphragm, intercostals, and lung tissue in response to 5-day CMV. Principal component analysis of metabolomic profiles in **(A)** diaphragm, **(B)** intercostals, and **(C)** lung tissue. **(D)** Venn diagram of all detected metabolites (AMs) among tissues. Volcano plot of differential metabolites (*p* < 0.05, absolute fold change ≥1.5) in response to 5-day CMV in **(E)** diaphragm, **(F)** intercostals and **(G)** lung tissue. **(H)** Venn diagram of differential metabolites (DMs) among tissues. Hierarchical clustering analysis of differential metabolites in **(I)** diaphragm, **(J)** intercostals and **(K)** lung tissue. **(L)** percentage of differential metabolites in all metabolites detected in each tissue. D0: rats without controlled mechanical ventilation (*n* = 5); D5: rats subject to controlled mechanical ventilation (*n* = 5 for diaphragm and intercostal; *n* = 4 for lung).

A total of 817, 786, and 941 metabolites were detected in diaphragm, intercostals, and lung tissue, respectively. The metabolomic profiles of diaphragm, intercostals, and lung of rats in response to 5 days CMV (D5) compared with 0-day sham operated rats (D0) are presented in Figure 1. The results of principal component analysis demonstrate significant separation between 5-day CMV and 0-day group in diaphragm (Figure 1A), intercostals (Figure 1B), and lung tissue (Figure 1C), demonstrating dramatic alterations in metabolomic profiles in all three tissues in response to 5-day CMV. A total of 680 metabolites were co-detected among all tissues (Figure 1D).

The subsequent differential analyses between 5-day CMV and 0-day sham controls identified a total of 242, 330, and 290 differential metabolites (DMs) in diaphragm, intercostals, and lung tissue, respectively, with a cutoff *p*-value < 0.05 and an absolute fold change ≥1.5 (Figures 1E–G; Supplementary File S1). In diaphragm, 111 DMs were increased and 131 DMs were decreased, with p-cresol glucuronide, trimethylamine N-oxide (TMAO), N-delta-acetylornithine, X-23639 (ID provided by Metabolon; unnamed metabolite), and stachydrine being the top-ranked DMs. In intercostals, 227 DMs were increased and 103 DMs were decreased, with TMAO, stachydrine, 1-linoleoyl-2-linolenoyl-GPC (18:2/18:3), X23639, and N-delta-acetylornithine being the top-ranked DMs. In lungs, 195 DMs were increased and 95 DMs were decreased with p-cresol sulfate, docosapentaenoylcarnitine (C22:5n3), docosatrienoylcarnitine (C22:3), stachydrine, and guaiacol sulfate being the top-ranked DMs. A total of 73 DMs were co-identified among all tissues, 75 DMs were uniquely identified in diaphragm, 127 DMs in intercostals, and 131 DMs in lung tissue (Figure 1H).

In addition, hierarchical clustering analyses of DMs (Figures 1I–K) showed distinct differences in metabolite profiles between D5 and D0 in all three tissues, validating the accuracy of differential analyses and confirming the strong impact of 5-day CMV on the metabolomic profiles in all three tissues. The percentage of DMs in all detected metabolites (AMs) in diaphragm, intercostals, and lung tissue were 29.6, 42, and 30.8% (Figure 1L). Thus, 5-day CMV had the strongest impact on the metabolomic profiles of intercostals, followed by lung and diaphragm.

### Classification of Differential Metabolites

**FIGURE 2:**
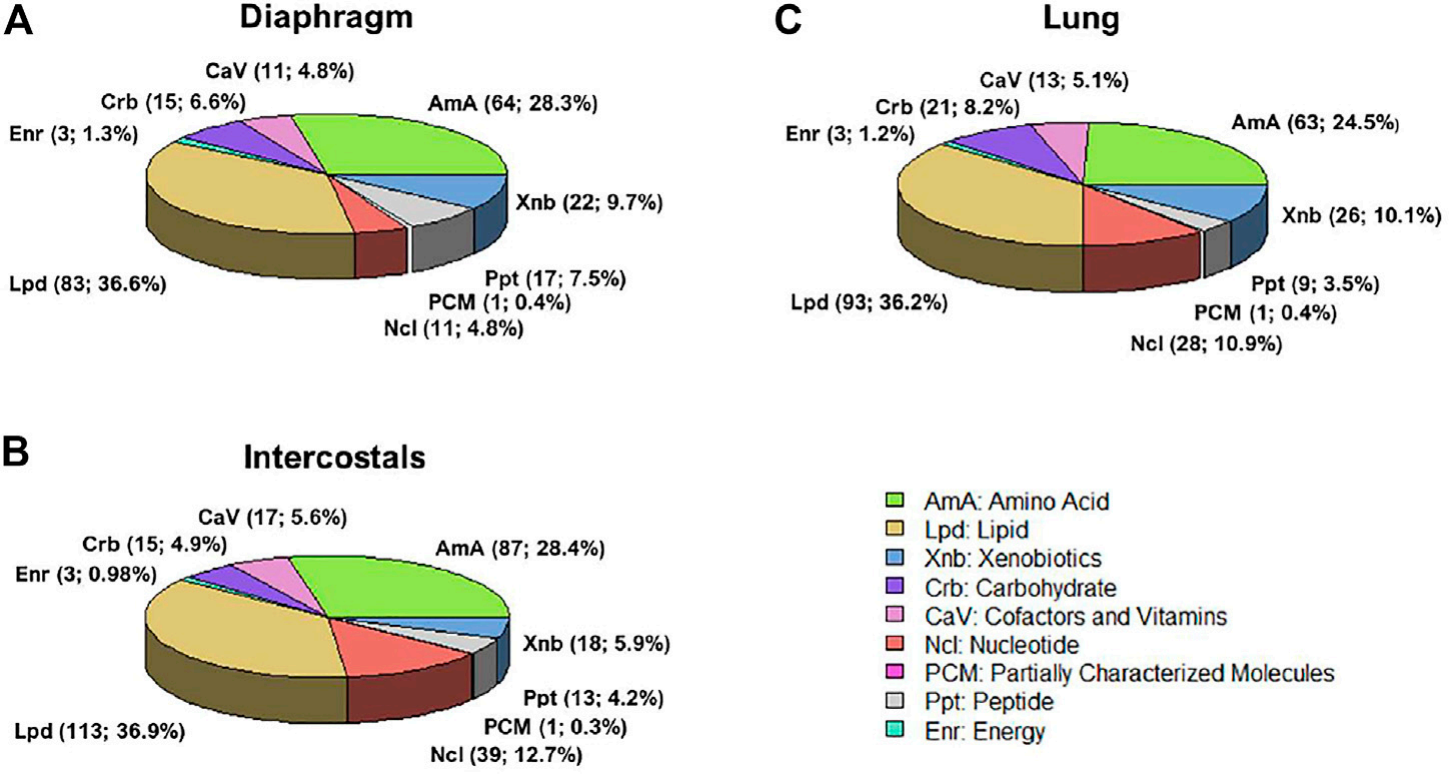
Differential metabolites classification in **(A)** diaphragm, **(B)** intercostals, and **(C)** lung tissue. According to the Metabolon annotation list, there are nine categories, including Amino Acid (AmA), Cofactors and Vitamins (CaV), Carbohydrate (Crb), Energy (Enr), Lipid (Lpd), Nucleotide (Ncl), Partially characterized molecules (PCM), Peptide (Ppt), Xenobiotics (Xnb).

According to the Metabolon annotation list, DMs were classified into nine categories, including Amino Acid, Cofactors and Vitamins, Carbohydrate, Energy, Lipid, Nucleotide, Partially characterized molecules, Peptide, Xenobiotics. In all tissues, the majority DMs belonged to the Lipid and Amino Acid category (Figures 2A–C).

### Metabolite-Metabolite Interaction Networks

**FIGURE 3:**
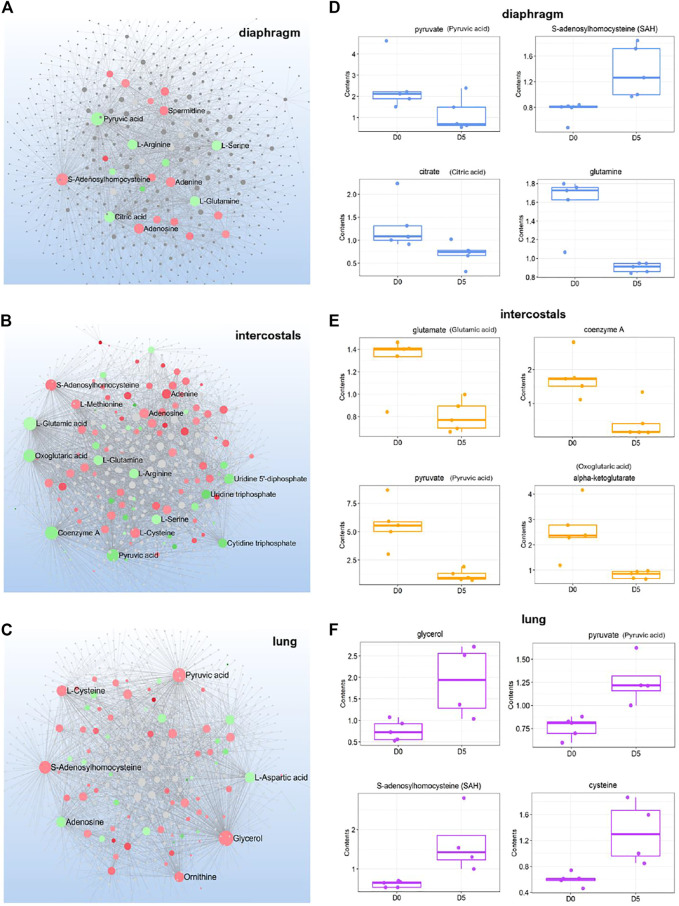
Metabolite-metabolite interactions network of differential metabolites **(A-C)** and boxplot for the 4 top-ranked metabolite hubs **(D-F)** in diaphragm, intercostals, and lung tissue. Metabolite hubs in the network are highlighted in colors, where green color indicates the downregulation and red color indicates the upregulation. Dot size represents the betweenness/association among metabolites.

To investigate the interactions among metabolites, DMs induced by 5-day CMV in each tissue were subject to metabolite-metabolite interaction analysis (Figures 3A–C). The most important metabolite hubs in diaphragm, intercostals, and lung tissue are highlighted in the MMI networks and the levels of the top 4 metabolite hubs are shown in Figures 3D–F. In diaphragm, the decreased DMs were annotated to energy metabolism (e.g., pyruvate, citrate) and amino acid metabolism (e.g., arginine, serine and glutamine), and the increased DMs were annotated to purine metabolism (e.g., adenosine and adenine). S-Adenosylhomocysteine (SAH) and spermidine were the most strongly interconnected metabolites. In intercostals, the decreased DMs were annotated to metabolites in energy metabolism (e.g., pyruvate, oxoglutaric acid, Coenzyme A), amino acid metabolism (e.g., glutamate, glutamine, arginine, serine), and pyrimidine metabolism (e.g., uridine 5′-diphosphate, uridine triphosphate, cytidine triphosphate), and the increased DMs were related to purine metabolism (e.g., adenine, adenosine), as well as SAH, methionine. In lung tissue, on the other hand, the increased DMs were annotated to energy metabolism (e.g., pyruvate, glycerol) and amino acid metabolism (e.g., cysteine, ornithine), and the decreased DMs were annotated to purine metabolism (e.g., adenosine). Some metabolite hubs were co-identified in all three tissues, such as SAH being increased in all three tissues, pyruvate levels were decreased in muscle tissues but increased in the lung tissue, and adenine and adenosine were increased in the muscles but decreased in the lung. These results show strong interactions among DMs indicating important biofunctions of the metabolite hubs in response to 5-day CMV.

### Metabolomic Pathway Enrichment Analysis

Metabolomic pathway enrichment analyses were performed to investigate the involvement of DMs in specific bioprocesses. Two enrichment analysis methods were employed in the present study: 1) enrichment analysis with combination of enrichment value (EV) and zscore and 2) metabolite set enrichment analysis (MSEA). Similar results were obtained independent on methods used (Supplementary File S2).

#### Metabolomic Pathway Signatures of Each Tissue in Response to 5-day CMV

The enriched pathways were considered as metabolomic pathway signatures in response to 5-day CMV when meeting the following strict cut-offs: absolute zscore ≥2.5, EV ≥ 1.5 and contained differential metabolites (DMs) ≥ 5. Zscore was the indicator used for the up-/down-regulated status of enriched pathways. Enriched pathways were evaluated by absolute (abs) zscore based on the directions of involved DMs and by EV based on the involved DMs/all detected metabolites (AMs) ratio.

**FIGURE 4:**
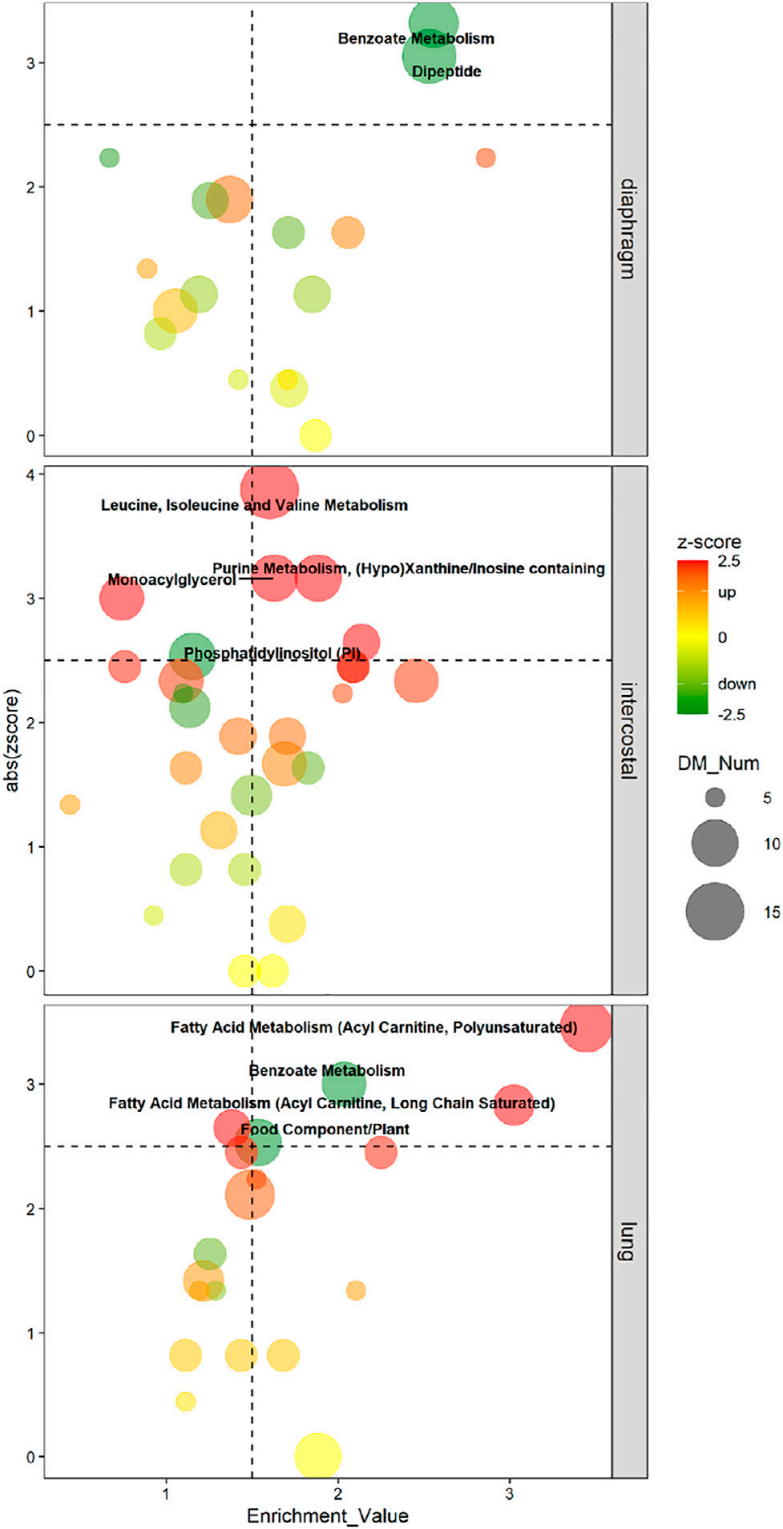
Four-Quadrant charts demonstrate the metabolomic pathway signatures of each tissue in response to 5-day CMV. X-axis represents the enrichment value of the pathway; Y-axis represents the absolute zscore of the pathway. Metabolic subpathway signatures is considered when enrichment value ≥1.5 and abs zscore ≥2.5. Color scale bar represents zscore which is used to predict the status of the pathway. Green color (negative zscore) indicates downregulation, whereas red color (positive zscore) indicates upregulation. Dot size represents the number of DMs in each pathway.

In diaphragm, “Benzoate Metabolism” (11 DMs; zscore: -3.32; EV: 2.56) and “Dipeptide” (13 DMs; zscore: −3.05; EV: 2.53) were the pathways with the lowest negative zscore and considered the metabolomic pathway signatures, i.e., DMs involved in “Benzoate Metabolism” and “Dipeptide” were significantly decreased in diaphragm’s response to 5-day CMV (Figure 4). In intercostals, the metabolomic pathway signatures were “Leucine, Isoleucine, and Valine Metabolism” (15 DMs; zscore: 3.87; EV: 1.60), i.e., the pathway with the highest positive zscore, followed by “Purine Metabolism, (Hypo)Xanthine/Inosine containing” (10 DMs; zscore: 3.16; EV: 1.88), “Monoacylglycerol” (10 DMs; zscore: 3.16; EV: 1.63), and “Phosphatidylinositol (PI)” (7 DMs; zscore: 2.65; EV: 2.14). These four pathways were significantly upregulated in intercostals in response to 5-day CMV (Figure 4). In lungs, “Fatty Acid Metabolism (Acyl Carnitine, Polyunsaturated)” (12; zscore: 3.46; EV: 3.44) was the pathway with the highest positive zscore followed by “Fatty Acid Metabolism (Acyl Carnitine, Long Chain Saturated)” (8 DMs; zscore: 2.83; EV: 3.02), and “Benzoate Metabolism” (9 DMs; zscore: -3; EV: 2.03), and “Food Component/Plant” (10 DMs; zscore: −2.53; EV: 1.53) were the pathways with the lowest negative zscores (Figure 4), demonstrating DMs involved in “Fatty Acid Metabolism (Acyl Carnitine, Polyunsaturated)” and “Fatty Acid Metabolism (Acyl Carnitine, Long Chain Saturated)” were significantly increased, whereas DMs involved in “Benzoate Metabolism” were significantly decreased in lung’s response to 5-day CMV.

#### Comparisons of Metabolic Pathways by MSEA

**FIGURE 5:**
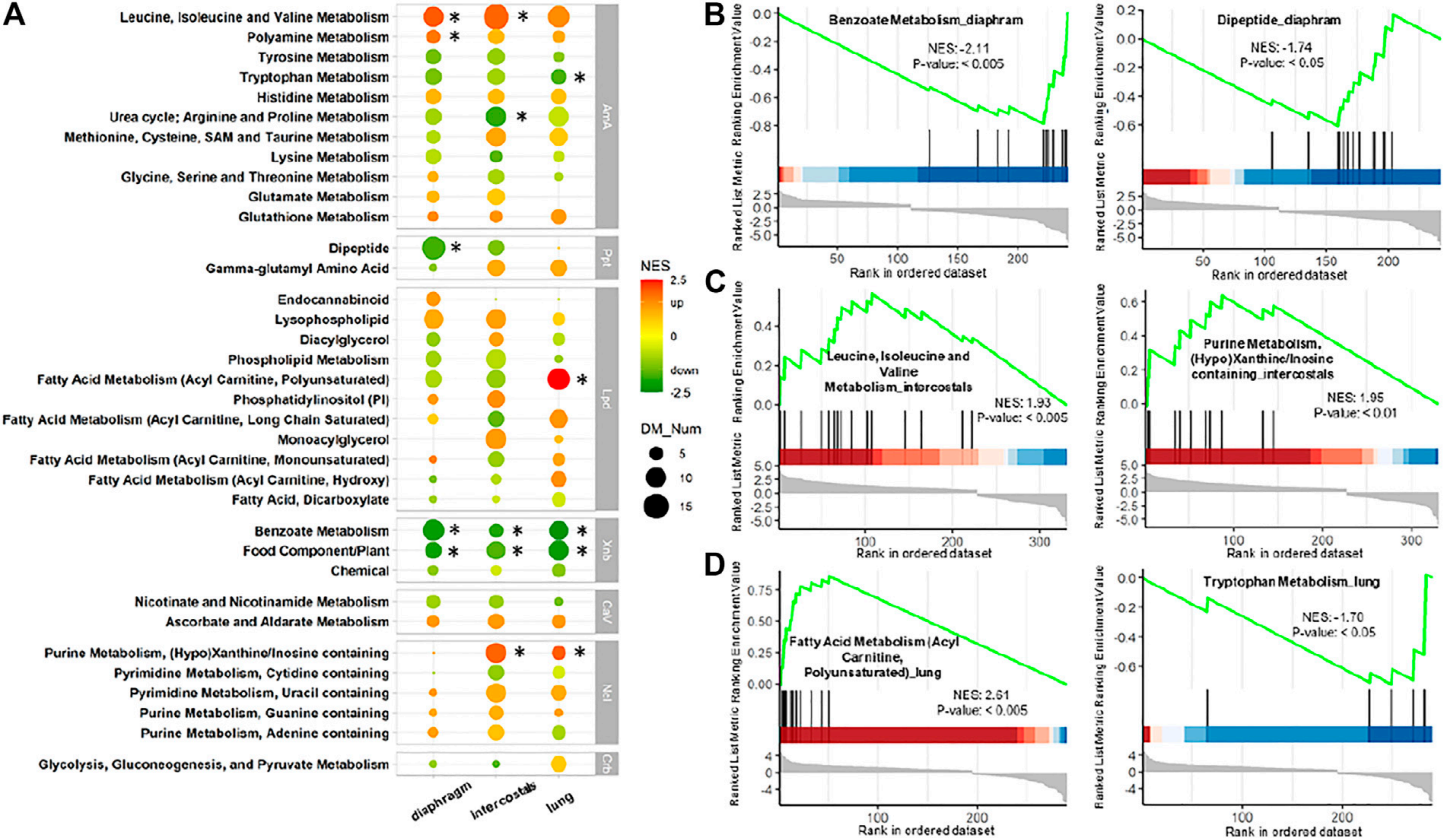
Metabolite set enrichment analysis (MSEA) of DMs in each tissue. **(A)** Comparison of enriched pathways among three tissues. Pathways are shown when contained DMs ≥5 in any tissue. Color scale bar represents normalized enrichment score (NES) which is the indicator of status of the enriched pathways. Green color (negative NES) indicates downregulation, whereas red color (positive NES) indicates upregulation. Dot size represents the number of enriched DMs. * Represents *p*-value < 0.05 and contained DMs ≥5. AmA: amino acid; CaV: cofactors and vitamins; Crb: carbohydrate; Lpd: lipid; Ncl: nucleotide; Ppt: peptide; Xnb: xenobiotics. MSEA-enrichment plot of the top-ranked enriched metabolites set in **(B)** diaphragm, **(C)** intercostals, and **(D)** lung tissue.

Enriched metabolic pathways are shown in Figure 5 when contained DM numbers were ≥5 in any of three tissues and pathways were significantly enriched when *p* < 0.05. Normalized enrichment score (NES) represents the indicator of the up-/down-regulated status of the enriched pathways. A higher positive NES indicates the pathway contains a higher percentage of increased DMs, and a lower negative NES means a higher percentage of decreased DMs.

The comparison of enrichment results among the three tissues revealed similarities and tissue-specific differences in response to 5-day CMV (Figure 5A). In the Xenobiotics category, the “Benzoate Metabolism” and “Food Component/Plant” were significantly enriched in all tissues with very low negative NES. In addition, “Benzoate Metabolism” was the most enriched pathway with the lowest negative NES in diaphragm (Figure 5B). In the Amino Acid category, “Leucine, Isoleucine and Valine Metabolism” were upregulated in all tissues and was significantly enriched in both diaphragm and intercostals (Figure 5C). “Tryptophan Metabolism” (Figure 5D) and “Urea cycle; Arginine and Proline Metabolism” were downregulated in all tissues but only significantly enriched in lung and diaphragm tissue. “Polyamine Metabolism” was significantly enriched only in diaphragm. In the Peptide category, tissue-specific differences were observed, such as “Dipeptide” being downregulated in the two muscle tissues, but only significantly enriched in diaphragm, and undetected in lung (Figure 5B). In the Nucleotide category, “Purine Metabolism, (Hypo)Xanthine/Inosine containing” (Figure 5C) were upregulated and significantly enriched in intercostals and lung tissue but undetected in diaphragm and it was the enriched pathway with the highest positive NES in intercostals. In the Lipid category, “Fatty Acid Metabolism (Acyl Carnitine, Polyunsaturated)” was upregulated and only significantly enriched in lung tissue but was downregulated in the other two tissues (Figure 5D) and it was the enriched pathway with the highest positive NES in lung tissue.

### DMs Involved in the Most Significantly Enriched Pathways

**FIGURE 6:**
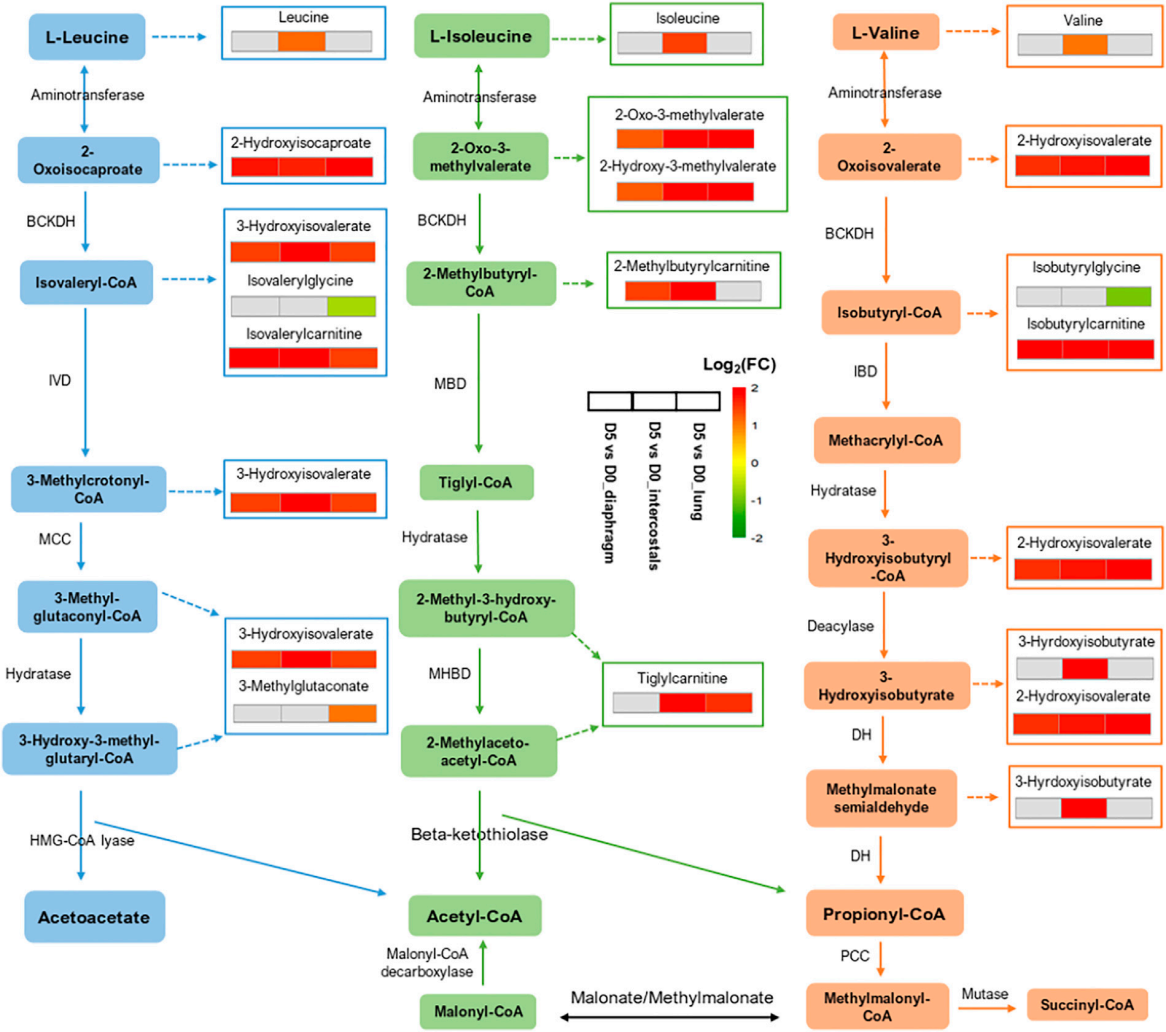
Illustration of “Leucine, Isoleucine and Valine Metabolism” and the involved DMs among tissues. Three adjacent blocks represent diaphragm, intercostals, and lung tissue (from left to right). Color scale bar represents Log_2_(FC).

**FIGURE 7:**
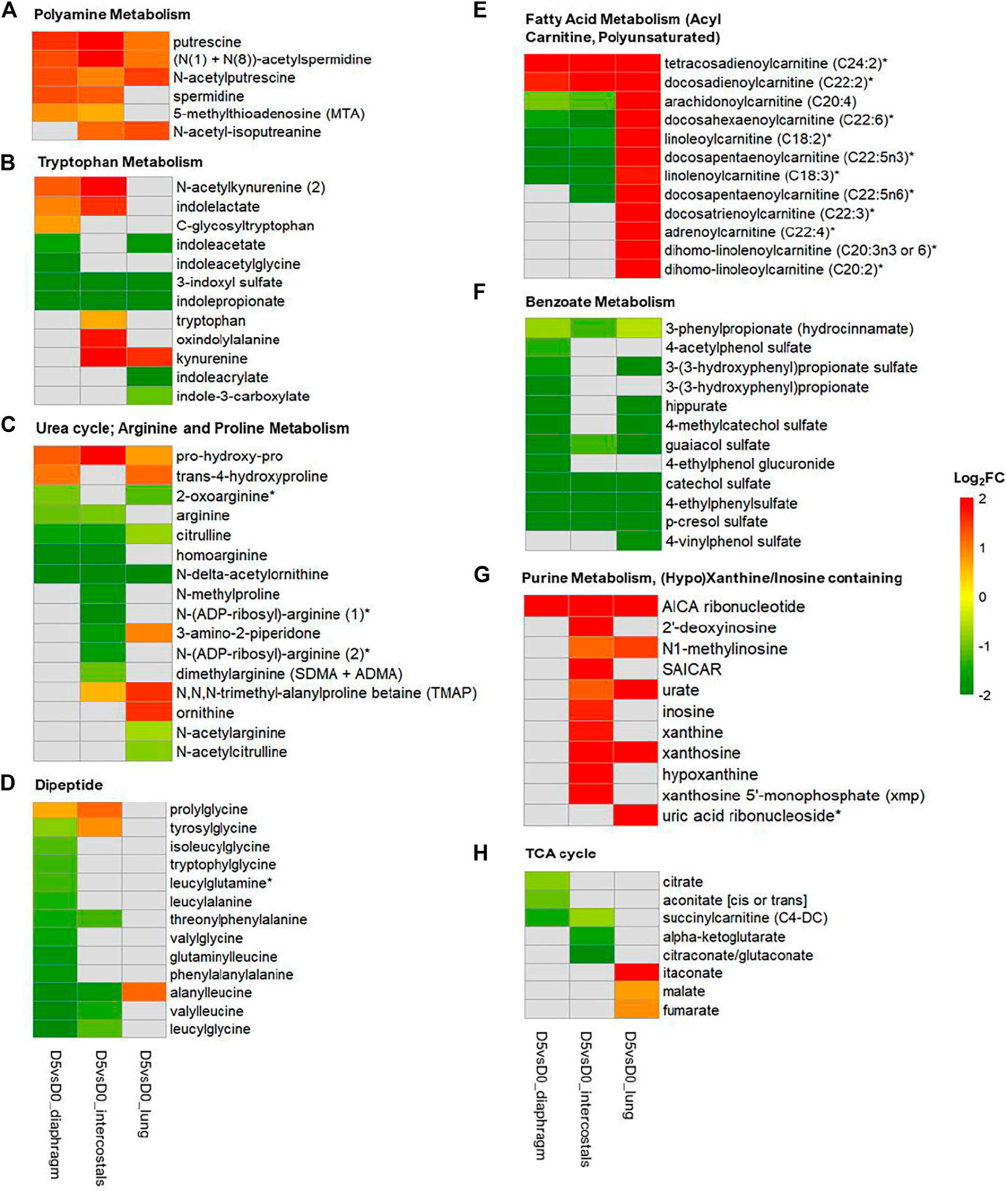
Heatmap of DMs involved in metabolomic signature pathways of all three tissues. **(A)** Polyamine Metabolism; **(B)** Tryptophan Metabolism; **(C)** Urea cycle; Arginine and Proline Metabolism; **(D)** Dipeptide; **(E)** Fatty Acid Metabolism (Acyl Carnitine, Polyunsaturated); **(F)** Benzoate Metabolism; **(G)** Purine Metabolism, (Hypo)Xanthine/Inosine containing; **(H)** TCA cycle. Color scale bar represents Log_2_(FC).

To visualize similarities and differences in significantly enriched pathways in the three tissues, the involved metabolites and their fold change information are shown in Figures 6, 7. In the Amino Acid category, all DMs involved in “Leucine, Isoleucine and Valine Metabolism” were increased in intercostals, including both the branch chain amino acids (BCAAs) and their catabolic metabolites. Similarity, all DMs were increased in diaphragm, although the levels of three BCAAs were not significantly altered. These results suggest increased levels of BCAAs degradation in the two respiratory muscles. In lung tissue, several catabolic metabolites of BCAAs were also increased but a few also decreased (Figure 6). The DMs involved in “Polyamine Metabolism” were increased in all tissues (Figure 7A). The DMs involved in “Tryptophan Metabolism” varied between tissues, and the majority DMs were decreased in lung tissue (Figure 7B). The majority DMs in “Urea cycle; Arginine and Proline Metabolism” were decreased in intercostals, whereas a smaller number of DMs were decreased in diaphragm and lung (Figure 7C). In the Peptide category, the majority of DMs in the “Dipeptide” was decreased in diaphragm, only a few in intercostals, and only one was identified as increased in lung tissue (Figure 7D). In the Lipid category, the number of DMs involved in “Fatty Acid Metabolism (Acyl Carnitine, Polyunsaturated)” was highest in lung tissue and all were increased, whereas fewer DMs were identified in two respiratory muscles and the majority being decreased (Figure 7E). In the Xenobiotics category, all DMs involved in “Benzoate Metabolism” were decreased in all tissues, with the highest number of DMs being observed in diaphragm, followed by lung and intercostals (Figure 7F). In the Nucleotide category, all the DMs involved in “Purine Metabolism, (Hypo)Xanthine/Inosine containing” were increased, with the highest number being observed in intercostals, followed by lung, and only one was identified in diaphragm (Figure 7G). DMs involved “TCA cycle” are also shown due to their essential role in energy metabolism, albeit with a smaller number of DMs (<5; Figure 7H).

## Discussion

Dramatic alterations in the metabolomic profiles were observed in respiratory muscles and lung after 5 days immobilization and mechanical ventilation. The majority of the DMs belonged to the Lipid and Amino Acid category. 5-day CMV showed the strongest impact on intercostals, followed by lung and diaphragm. Metabolite and metabolic pathway signatures were identified in each tissue based on differential analysis, MMI network analysis, and metabolic pathway enrichment analysis. The diaphragm, was characterized by more decreased DMs of “Dipeptides” while more increased DMs of “Leucine, Isoleucine and Valine Metabolism” and “Purine Metabolism, (Hypo)Xanthine/Inosine containing” were observed in intercostals. Lung, on the other hand, was characterized by more increased DMs of “Fatty Acid Metabolism (Acyl Carnitine; Polyunsaturated)”.

### Metabolite Alterations Related to Protein Degradation in Respiratory Muscles

We have previously reported dramatic muscle wasting of limb and trunk muscles in response to long-term CMV (Ochala et al., 2011; Corpeno et al., 2014; Salah et al., 2016). In accordance with previous transcriptomic analyses, metabolite alterations were associated with an accelerated degradation of myofibrillar proteins in response to immobilization and CMV such as augmented levels of branch-chain amino acids (BCAAs; leucine, isoleucine, and valine) and their catabolic metabolites in both diaphragm and intercostals (Figures 5A, 6), since myofibrillar proteins are composed of 18% BCAAs (Baracos and Mackenzie, 2006; Chiocchetti et al., 2021). DMs involved in “Dipeptide” were significantly decreased, especially in the diaphragm (Figures 5A, 7D). The reduction of dipeptides may also contribute to an accelerated protein breakdown since they protect from protein degradation (Solomon et al., 1998; Tasaki and Kwon, 2007). According to differential analyses, trimethylamine N-oxide (TMAO), one of the five top-ranked DMs, showed decreased levels in both diaphragm and intercostals (Figures 1E,F). TMAO exhibits stabilizing effects on proteins, including heavy meromyosin and actomyosin complexes, although underlying mechanisms of action remain incompletely understood (Kumemoto et al., 2012; Ma et al., 2014). The reduction of TMAO may accordingly play a role in the breakdown of the myofibrillar proteins in the respiratory muscles; contributing to the muscle dysfunction and muscle loss associated with VIDD and CIM.

### Metabolite Alterations Related to Inflammatory Responses

Long-term CMV has detrimental effects on lung, referred to as the ventilator-induced lung injury (VILI). Based on clinical and experimental studies, we have hypothesized that VILI and the subsequent release of cytokines and chemokines may induce systematic inflammation affecting peripheral organs including respiratory and limb muscles (Lyu et al., 2021; Cacciani et al., 2022). This is further supported by current metabolomic alterations related to inflammatory responses in diaphragm, intercostals, and lung tissue.

During acute and chronic inflammation, arginine is converted to polyamines including putrescine, spermidine, and spermine. These polyamines exhibit anti-inflammatory effects due to their anti-oxidant and/or lysosomal stabilization properties (Satriano, 2004; Lagishetty and Naik, 2008). The reduced level of arginine (diaphragm and intercostals) but augmented levels of polyamines in all tissues support an active ongoing inflammatory response (Figure 5A, 7A, 7C). On the other hand, during inflammation, tryptophan metabolism is dysregulated and most likely converted to kynurenine (Wang et al., 2015) rather than degraded into metabolites involved in “Tryptophan Metabolism” (Figure 7B) and aromatic metabolites like those involved in “Benzoate Metabolism” (Figure 7F), being triggered by proinflammatory stimuli (e.g., interferon-γ, tumor necrosis factor-α) (Cervenka et al., 2017). Kynurenine participates in and contributes to immune activities *via* its regulation on T cells (Sorgdrager et al., 2019). Thus, the augmented levels of kynurenine observed in intercostals and lung tissue and reduction of metabolites involved in “Benzoate Metabolism” and “Tryptophan Metabolism” in all tissues are forwarded as an active inflammatory response to 5-day CMV. In addition, inflammatory response-induced cell lysis, apoptosis, and degranulation result in the release of purines-abundant metabolites such as ATP, NAD+, and nucleic acids, contributing to the augmented level of DMs involved in “Purine Metabolism” (Figure 7G) in intercostals and lung tissue (Matsumoto et al., 1979; Linden et al., 2019). Thus, cell lysis and apoptosis were more severe in intercostal muscle than diaphragm which may be protected by the mechanical loading induced by the ventilator and the concomitant inhibition of apoptosis (Corpeno Kalamgi et al., 2016).

According to MMI network analysis, SAH was the metabolite hub increased in all tissues (Figure 3). The increased level of or accumulation of SAH may have a role in inflammatory responses *via* activation of the NFκB signaling pathway. SAH is the product of almost all methylation reactions involving S-Adenosylmethionine (SAM) as methyl donor *in vivo*. The alterations in SAH may have effects on epigenetic modification and gene expression pattern. SAH is a potent inhibitor for SAM-dependent methyltransferases, such as the enhancer of Zester homolog 2 (EZH2). As the target of EZH2, the trimethylation of lysine 27 on histone 3 (H3K27me3) is the major mark of transcriptional repression in mammalian cells. The accumulation of SAH may inhibit the activity of EZH2, resulting in decreased H3K27me3, which in turn removes the suppression of the expression of several genes, including *IL1B*. By stimulating its receptor, IL1β can contribute to NFκB activation (Barroso et al., 2016). Stachydrine (also named proline betaine belonging to proline and derivatives category) was identified as the top-ranked DMs and decreased levels were observed in all tissues (Figures 1E–G). Just like other secondary metabolites, stachydrine is a metabolically and physiologically non-essential metabolite, but acts as a signaling molecule and it has been reported to attenuate IL-1β-induced inflammatory response *via* acting on the NF-kB signaling pathway (Li et al., 2020; Wu et al., 2020) and the reduced stachydrine levels may contribute to inflammatory responses in all tissues after 5-day CMV.

### Metabolite Alterations Related to Energy Metabolism

According to transcriptomic analyses of the diaphragm in rats exposed to 5-day immobilization and CMV, genes related to energy production like glycolysis, TCA cycle and oxidative phosphorylation were downregulated (Lyu et al., 2021). In the current study, many metabolite alterations confirmed attenuated energy metabolism in respiratory muscles after 5-day immobilization and CMV. According to MMI network analyses in diaphragm and intercostals, decreased levels of metabolite hubs like pyruvate were observed, as well as decreased levels of citrate and coenzyme A linked to the TCA cycle (Figures 3A,B,D,E). Metabolites involved in “Glycolysis, Gluconeogenesis, and Pyruvate Metabolism” were also observed decreased (Figure 5A). These results suggest attenuated energy metabolism and ATP production in the respiratory muscles’ response to the immobilization caused by post-synaptic blockade of neuromuscular transmission.

In lung tissue, metabolite hubs like pyruvate changed in an opposite direction compared with diaphragm and intercostals (Figures 3C,F). The increased glycerol (Figure 3C) level suggests degradation of triglycerides into free fatty acid, followed by the upregulation of “Fatty Acid Metabolism” (Figures 5A, 7E) and combined with the enhanced “TCA cycle” (Figure 7H), reflecting an increased energy production.

In conclusion, the metabolomic profiles of diaphragm, intercostals, and lung tissue were dramatically affected by long-term immobilization and CMV, i.e., the ICU condition. The alterations in metabolomic profiles may represent the end metabolic products of several biological processes including an accelerated myofibrillar protein degradation in diaphragm and intercostals, active inflammatory responses in all tissues, and enhanced energy production in the lung but attenuated in the two respiratory muscles. The identified metabolite and metabolic pathway signatures contribute to an improved understanding of mechanisms underlying VILI, VIDD, and CIM and the discovery of biomarkers which need to be extended to clinical studies in mechanically ventilated ICU patients. In ongoing experiments, interventions targeting VILI by systemic administration of human bone marrow derived mesenchymal stromal cells or extracellular vesicles derived from these cells are investigated in rats exposed to long-term CMV. Preliminary results show significant improvement of force generating capacity and reduced muscle fiber atrophy in both diaphragm and limb muscle fibers in response to these interventions in long-term mechanically ventilated rats.

## Data Availability

The original contributions presented in the study are included in the article/Supplementary Material, further inquiries can be directed to the corresponding author.
